# The Active Microbiota of the Eggs and the Nauplii of the Pacific Blue Shrimp *Litopenaeus stylirostris* Partially Shaped by a Potential Vertical Transmission

**DOI:** 10.3389/fmicb.2022.886752

**Published:** 2022-05-12

**Authors:** Carolane Giraud, Nolwenn Callac, Viviane Boulo, Jean-Sébastien Lam, Dominique Pham, Nazha Selmaoui-Folcher, Nelly Wabete

**Affiliations:** ^1^UMR 9220 ENTROPIE, Ifremer (LEAD-NC), Noumea, New Caledonia; ^2^Institut des Sciences Exactes et Appliquées (ISEA), University of New Caledonia, Noumea, New Caledonia

**Keywords:** active microbiota, shrimps, eggs, nauplii, vertical transmission, microbial colonization, core microbiota

## Abstract

The many ecological niches present in an organism harbor distinct microorganisms called microbiota. Different factors can influence the establishment of these commensal microbial communities. In a previous article, we have concluded that some bacterial lineages associated with the early larval stages of the Pacific blue shrimp *Litopenaeus stylirostris* could be acquired from the breeders *via* a potential vertical transmission. The present study was conducted in order to investigate this hypothesis. Using HiSeq sequencing of the V4 region of 16S rRNA gene, we analyzed the active microbiota associated with the eggs and the nauplii of *L. stylirsotris* as well as with the reproductive organs of their breeders. Microbial communities associated with the rearing water were also considered to discriminate environmental microbial lineages. Using these analyses, we highlight a set of core bacterial families present in all samples and composed of members of *Colwelliaceae*, *Alteromonadaceae*, *Pseudoalteromonadaceae*, *Saccharospirillaceae*, *Oceanospirillaceae*, *Vibrionaceae*, *Burkholderiaceae*, *Rhodobacteraceae*, *Flavobacteraceae*, and *Corynebacteriaceae*; showing the importance of the environment in the establishment of the larval microbiota. We also present specific bacteria affiliated to the *Arcobacteraceae*, *Rhodobacteraceae*, *Comamonadaceae*, and *Colwelliaceae* families, which were only found in the breeders and their offspring strengthening the hypothesis of a potential vertical transmission shaping the active microbiota of the eggs and the nauplii of *L. stylirostris*.

## Introduction

In animals and plants, temperature, pH, and oxygen levels as well as nutrient accessibility vary throughout the organism resulting in many different available ecological niches (Ikeda-Ohtsubo et al., 2018). These contrasting environments can be colonized by various and distinct microorganisms. Microorganisms associated with organs covered by epithelial cells and in contact with the external environment of the host (gut, skin, oral cavity, urogenital, and respiratory tracts) are referred to as commensal microorganisms (Tlaskalová-Hogenová et al., 2004; Aas et al., 2005; Whiteside et al., 2015; Ikeda-Ohtsubo et al., 2018). All the microbial taxa inhabiting a given environment in a host form a microbiota (Ursell et al., 2012). In all organisms, the microbiota is known to have great importance in the health status of the host as it plays a key role in digestion, immunity, nutrient intake, development, and behavior (Cho and Blaser, 2012). In addition, studies have reported that microbiota dysbiosis is often linked to disease outbreak (Egan and Gardiner, 2016). Even though evidence is mounting on the essential role of the microbiota, very little is known about the establishment of these commensal microbial communities in a given tissue. Some studies suggest that environmental factors shape the microbiota. For example, studies have shown that diet influences the microorganisms associated with the gut of humans, fish, shrimps, and crabs (Nayak, 2010; Ursell et al., 2012; Sun and Xu, 2021). However, other factors can also be involved in the modulation of the microbiota such as host selection pressure and genetic background (Li et al., 2012; Sun and Xu, 2021). Interestingly, commensal microorganisms can also be transmitted from one generation to another *via* a vertical transmission. In humans, studies have shown that the microbiota of infants was similar to the vaginal microbiota of their mother after natural birth but mimicked the skin microbiota when caesarean section was performed (Cho and Blaser, 2012; Ursell et al., 2012). Vertical transmission of bacteria has also been suggested in marine organisms such as fish and sponges and has been experimentally proved in salmons (Larenas et al., 2003; Lee et al., 2009; Ikeda-Ohtsubo et al., 2018; Nyholm, 2020). Furthermore, mother to offspring bacterial transmission has been strongly hypothesized to modulate the microbiota of the eggs of the vent shrimp *Rimicaris exoculata* (Guri et al., 2012; Methou et al., 2019).

In a previous study that we conducted, we have also suggested that some bacterial strains could be vertically transmitted in the Pacific blue shrimp *Litopenaeus stylirostris* (Giraud et al., 2021). Indeed, studying the active microbiota associated with the early larval stages (eggs and nauplii) of *L. stylirostris* as well as with their rearing environment, several bacterial families were specifically associated with the eggs and the nauplii and were not found in the environment leading us to hypothesize that they had been acquired from the breeders. In order to determine if microbial vertical transmission is indeed involved in the establishment of commensal microbial communities in *L. stylirostris*, we present a new study focusing on the active microbiota of the eggs and the nauplii of *L. stylirostris*. We also investigate the microbial communities associated with their rearing environment as well as with the female and male reproductive organs of their breeders. We highlight a core microbiota associated with all the samples showing the importance of environmental factors in the establishment of commensal microorganisms in the animals. We also identify several bacterial families specific to the breeders and their offspring strengthening our hypothesis of a vertical transmission in *L. stylirostris*. Some specific bacterial lineages associated with the eggs and the nauplii also suggest the influence of other underestimated factors. Nevertheless, this study brings insight on the establishment of the active microbiota associated with the first larval stages of a farmed shrimp species.

## Materials and Methods

### Study Design and Sample Collection

**Figure 1:**
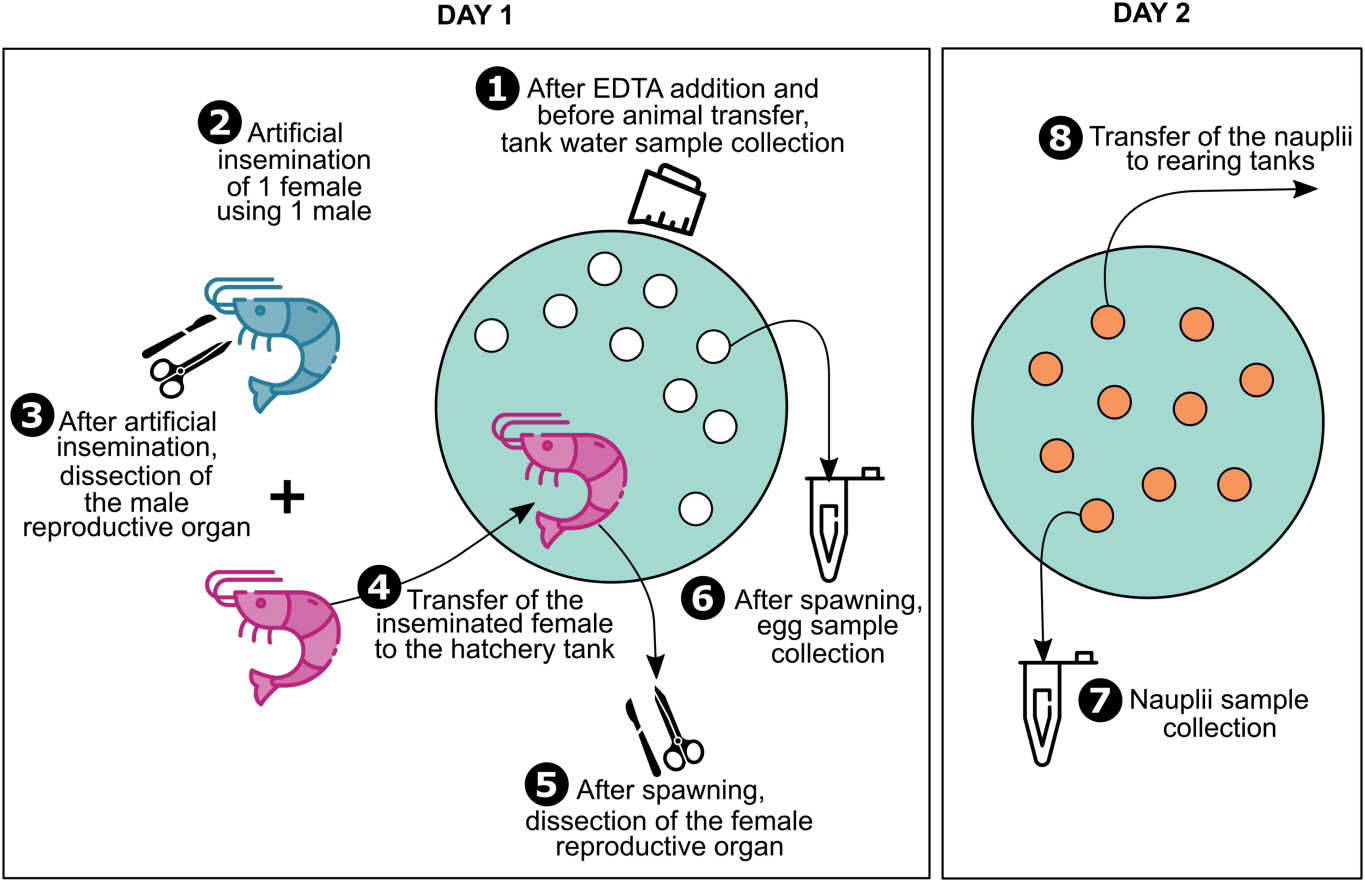
Sample collection. Sample collection was performed using an eight-steps technique. First, water was sampled from the hatchery tanks after ethylenediaminetetraacetic acid (EDTA) addition. Then, on the day the tanks were filled and sampled, artificial inseminations were performed. In order to consider two-parent families only, one male was used to inseminate one female for all the artificial inseminations. After artificial inseminations, males were dissected in order to sample their reproductive organ and females were transferred into the hatchery tanks at a rate of 1 individual per tank and were left to spawn. After spawning, females were retrieved to dissect their ovarian tissue, and eggs were sampled from all the hatchery tanks. To allow larval hatching, eggs were left until the next day in the hatchery tanks. Before transfer into rearing tanks, the nauplii were sampled from each tank. A total of three two-parent families were considered for this study.

Eggs and nauplii of *L. stylirostris* were supplied by the experimental shrimp hatchery located at the Saint Vincent Bay (Ifremer, Boulouparis, New Caledonia) in May 2021. Breeders were reared in earthen ponds before transfer to maturation tanks according to the method described in Pham et al. (2012). Tanks in the maturation and in the hatchery were filled with treated natural seawater from the Saint Vincent Bay as described in Giraud et al. (2021). Water from three different hatchery tanks was sampled after addition of ethylenediaminetetraacetic acid (EDTA; Figure 1). For each tank, 1 L of seawater was filtered on a 0.2 μm pore size filter (S-PAK membrane filter, Millipore) and filters were stored at −80°C until further RNA extractions.

In order to study two-parent families, artificial inseminations were conducted using one male to inseminate one female on the day the hatchery tanks were filled and sampled. After artificial inseminations, females were transferred into the hatchery tanks at a rate of 1 individual per tank and left to spawn (Figure 1). Males were dissected using sterilized tweezers and scissors to sample their reproductive organs (testicular diverticula). After spawning, females were retrieved and dissected in the same way as the males in order to collect their ovarian tissues. All the collected reproductive organs were rinsed with sterilized seawater and stored in 2 ml sterile microtubes. After spawning, around a hundred eggs from each tank were collected in a 2 ml sterile microtube using sterilized pliers. The following day, before transfer into rearing tanks for larval rearing, nauplii were separated from non-hatched eggs and abundantly rinsed. Around a hundred nauplii were sampled from each tank in the same way as the eggs. All the collected samples were stored at −80°C until further RNA extractions.

A total of three two-parent families were considered for this study. For each family unique samples of male and female reproductive organs, eggs, and nauplii were collected (Figure 1). All water samples collected from the hatchery tanks as well as male and female reproductive organs are respectively called “Tank,” “Male,” and “Female” in the rest of the article. Egg and nauplius (nii) samples are referred to as such in the text. All sample names are followed by a number ranging from 1 to 3. Samples with the same number belong to the same two-parent family.

### RNA Extractions and Sequencing

In order to study the active microbial communities (Yates et al., 2021) associated with all the considered samples, RNA extractions were performed using the RNeasy PowerWater kit (Qiagen) for the filters, the eggs and the nauplii; and using the RNeasy minikit (Qiagen) for the male and the female reproductive organs. All the extracted RNAs were reverse-transcripted into complementary DNAs (cDNAs) using a two-step technique. The first step consisted in adding 200 ng of RNAs to a reaction mix [buffer 5X, dNTP 10 mM, random hexamers 50 μM, reverse transcriptase M-MLV (PROMEGA) 200u.μl^−1^, RNAse/DNAse free water] and performing reverse transcription in a thermocycler during 10 min at 25°C, 2 h at 42°C, and 5 min at 85°C. The second step was performed using the Second Strand cDNA Synthesis kit (ThermoFisher).

All the double-stranded cDNAs obtained were sent to MrDNA (Shallowater, TX, United States) in order to amplify and sequence the V4 region of the 16S rRNA gene using the 515F/806R primers (Caporaso et al., 2011). An HiSeq Illumina sequencing was conducted using a 2 × 300 pb paired end run and an average sequencing depth of 50 K raw reads per sample.

### Microbiota Analysis

Raw sequences were treated using the DADA2 package under RStudio (Callahan et al., 2016; RStudio Team, 2020) as described in Giraud et al. (2021). After the obtention of the ASV table, sequences affiliated to eukaryotas, chloroplasts, and mitochondrias were removed before further analysis.

A dendrogram based on a Bray–Curtis dissimilarity matrix and Ward method was constructed using the vegan package in RStudio (Oksanen et al., 2020). Histogram tables and diversity indexes were obtained using the phyloseq package in Rstudio (McMurdie and Holmes, 2013). Significative differences among diversity indexes were evaluated performing a Kruskal-Wallis test and a Dunn post-test. Venn diagrams were made using the open-source component for web environment jvenn (Bardou et al., 2014).^1^ Core microbiotas were determined using the constructed Venn diagrams based on shared membership of ASVs (Shade and Handelsman, 2012).

All the 16S rRNA data are available in the NCBI SRA repository under the BioProject PRJNA736535 (SRA accession numbers from SRR17301487 to SRR17301501).

## Results

### Total Microbial Diversity of all the Samples

A total of 5,409,237 reads were obtained from the HiSeq Illumina sequencing of all samples. Sequences were clustered into 2,344 ASVs. The smallest and largest libraries were respectively composed of 109,259 and 955,820 reads and respectively corresponded to the Egg_3 and Nii_1 (nauplii) samples.

**Figure 2:**
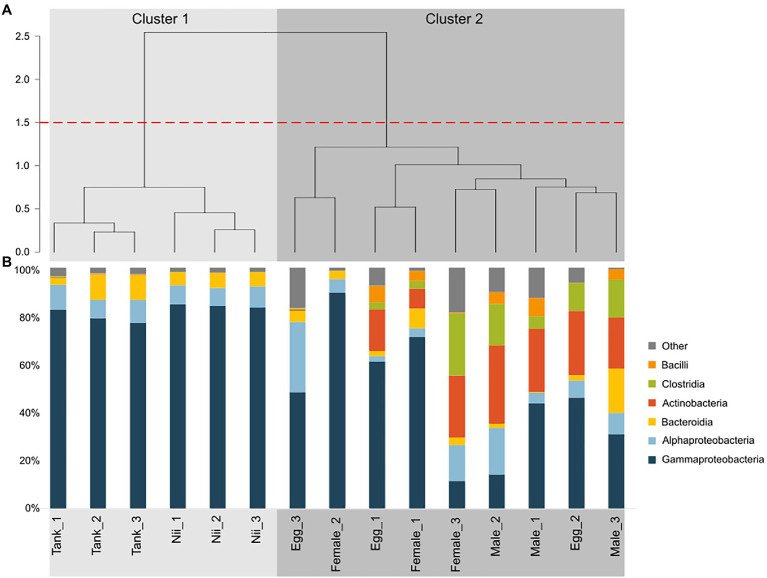
Clustering and total microbial composition of all the samples. **(A)** Hierarchical clustering based on Bray–Curtis dissimilarity and Ward method. Clusters were defined using a 1.5 threshold depicted by the red dotted line. All the tank water and the nauplius (nii) samples are gathered in Cluster 1, in light gray. All the female, the male and the egg samples are clustered in Cluster 2, in dark gray. **(B)** Bacterial compositions of the tank water, the nauplius (nii), the egg, the female and the male samples. Major bacterial classes were defined as the bacterial classes with a total relative abundance higher than 1%.

Hierarchical clustering based on Bray–Curtis dissimilarity separated the samples into two distinct groups (Figure 2A). The first cluster (Cluster 1) gathered all the tank water and the nauplius samples, while the second cluster (Cluster 2) grouped all the breeder and the egg samples. For all samples, six dominant bacterial classes with a total relative abundance higher than 1% were highlighted (Figure 2B). *Gammaproteobacteria* and *Alphaproteobacteria*, respectively, accounted for 72 and 8% of the total relative abundance. *Bacteroidia* and *Actinobacteria* each represented 6% of the ASV table. Finally, *Clostridia* and *Bacilli*, respectively had a total relative abundance of 3 and 1%. The tank waters and the nauplii showed consistent total microbial compositions with high proportions of *Gammaproteobacteria* as well as *Alphaproteobacteria* and *Bacteroidia*. All the male samples also had homogeneous microbial communities but with larger proportions of *Actinobacteria* and *Clostridia* compared to the water and nauplii. Unlike the other samples (water, nauplii, and males), the females and the eggs displayed important inter-individual variabilities. Indeed, the Egg_2 and the Female_3 samples had similar compositions to the male samples with a microbial diversity mainly composed by *Clostridia*, *Actinobacteria*, and *Proteobacteria*; whereas the other female and egg samples showed higher abundances of *Proteobacteria*.

### Shared ASVs Among Sample Types

**Figure 3:**
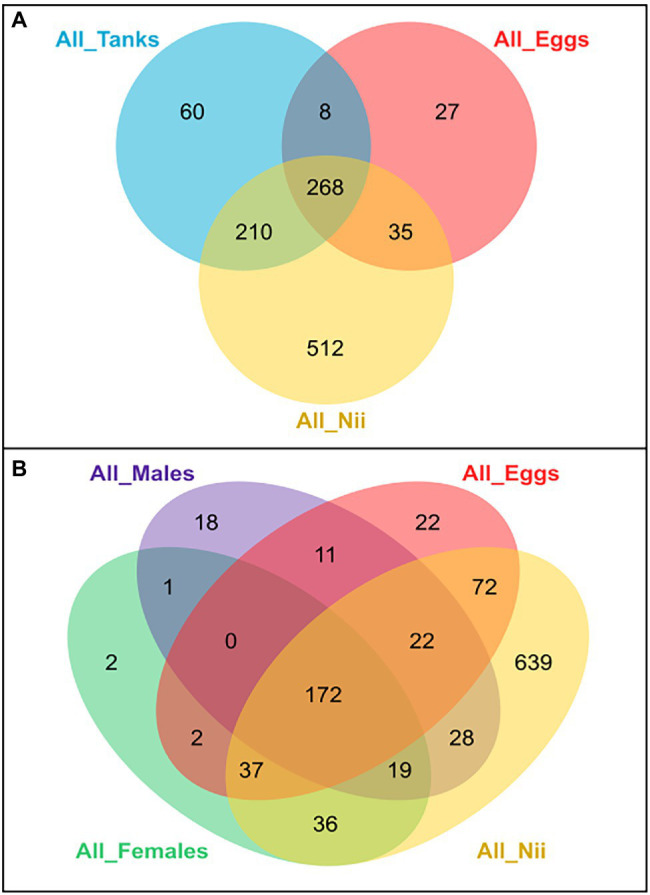
Global Venn diagrams of shared and specific ASVs among sample types. **(A)** Venn diagram of specific and shared ASVs among the tank water, the egg and the nauplius (nii) samples. The blue ellipse represents the ASVs shared by all the hatchery tank water samples. The red ellipse represents the ASVs shared by all the egg samples. The yellow ellipse represents the ASVs shared by all the nauplius (nii) samples. **(B)** Venn diagram of specific and shared ASVs among the breeders (female and male), the egg and the nauplius (nii) samples. The green ellipse represents the ASVs shared by all the female reproductive organ samples. The purple ellipse represents the ASVs shared by all the male reproductive organ samples. The red ellipse represents the ASVs shared by all the egg samples. The yellow ellipse represents the ASVs shared by all the nauplius (nii) samples. In both Venn diagrams, numbers noted in the overlapping areas correspond to the number of shared ASVs among sample types, while numbers noted outside of the overlapping areas correspond to the numbers of specific ASVs associated with each sample type.

Several Venn diagrams were constructed in order to highlight shared ASVs among the five considered sample types: eggs, nauplii, tank waters, female ovarian tissue, and male reproductive organs (Supplementary Figure S1). All the tanks shared 546 ASVs which represented 96% of the total relative abundance of all the water samples (Supplementary Figure S1A). The female reproductive organs displayed a core microbiota composed of 269 ASVs (86%; Supplementary Figure S1B). A total of 271 ASVs (83%) were shared among the male reproductive organs (Supplementary Figure S1C). Finally, the egg (Supplementary Figure S1D) and the nauplius (Supplementary Figure S1E) samples respectively shared 338 (85%) and 1,025 ASVs (99.5%). These five ASV lists were used to build two global Venn diagrams (Figure 3). The first global Venn diagram showed the specific and shared ASVs among the tank water, the egg and the nauplius samples (Figure 3A), whereas the second Venn diagram focused on the breeders and their offspring (Figure 3B).

### Shared Bacterial Lineages Among all the Samples

**Figure 4:**
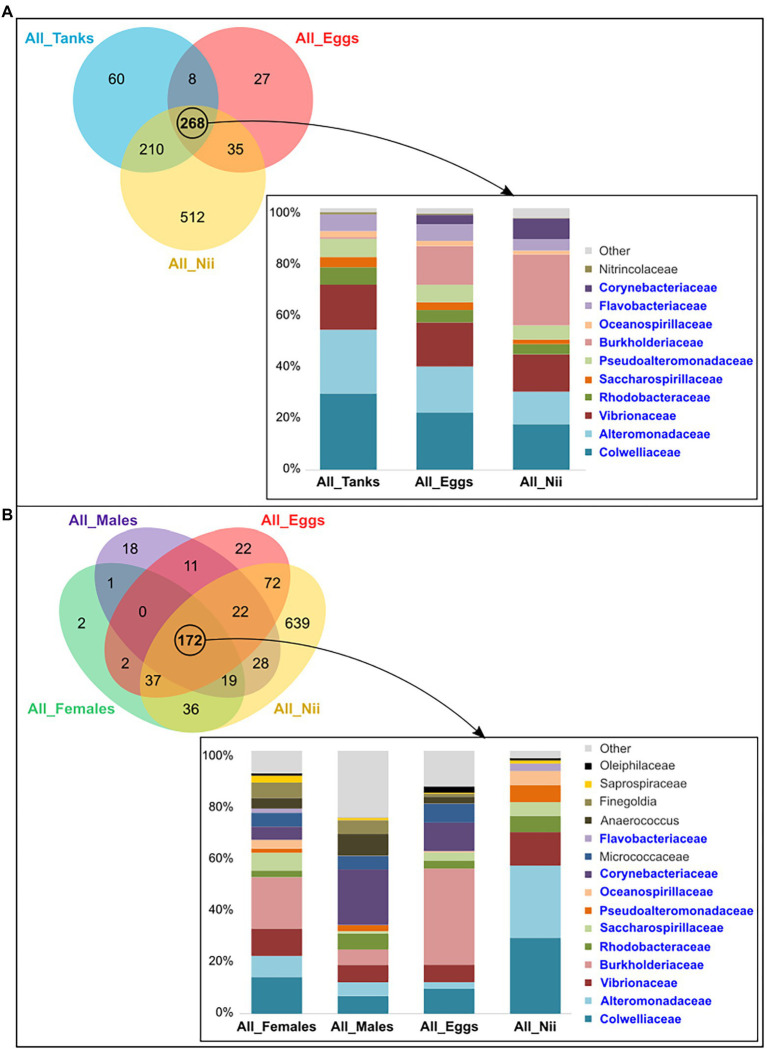
Shared bacterial lineages among the eggs, the nauplii, the tank waters, and the reproductive organs of the breeders. Top bacterial families with a total relative abundance higher than 1% shared among **(A)** the tank water, the egg, and the nauplius (nii) samples (268 ASVs) and **(B)** the female and the male reproductive organs, the egg and the nauplius (nii) samples (172 ASVs). The bacterial families in blue are those shared between the two histograms which were constructed by averaging the triplicates for each sample type.

The tank water, the egg and the nauplius samples shared 268 ASVs among which 11 dominant bacterial families with a total relative abundance higher than 1% were highlighted (Figure 4A). Comparing the microbial compositions of these samples, *Burkholderiaceae* seemed to be more abundant as the larvae grew, while the proportions of *Colwelliaceae* and *Alteromonadaceae* decreased. Furthermore, the abundance of *Vibrionaceae* was constant in the tank waters, the eggs, and the nauplii (Figure 4A). The eggs and the nauplii also shared 172 ASVs with the male and the female reproductive organs. When considering this subset of ASVs, 15 dominant bacterial families were highlighted (Figure 4B). The distribution profiles of these families varied between sample types. Indeed, the female and the male reproductive organs as well as the eggs showed higher abundances of *Burkholderiaceae*, *Corynebacteriaceae*, and *Micrococacceae* compared to the nauplii. On the contrary, abundances of *Colwelliaceae*, *Alteromonadaceae*, and *Pseudoalteromonadaceae* increased in the nauplius samples (Figure 4B). Comparing the two histograms, 10 families were shared between the two sets of core microorganisms: *Colwelliaceae*, *Alteromonadaceae*, *Vibrionaceae*, *Rhodobacteraceae*, *Saccharospirillaceae*, *Pseudoalteromonadaceae*, *Burkholderiaceae*, *Oceanospirillaceae*, *Flavobacteriaceae*, and *Corynebacteriaceae*. These bacterial lineages respectively represented 97 and 86% of the total relative abundance of the ASVs shared among the tank waters, the eggs and the nauplii (Figure 4A) and among the breeders and their offspring (Figure 4B).

### Shared Bacterial Lineages Among the Eggs, the Nauplii, and the Breeders

**Figure 5:**
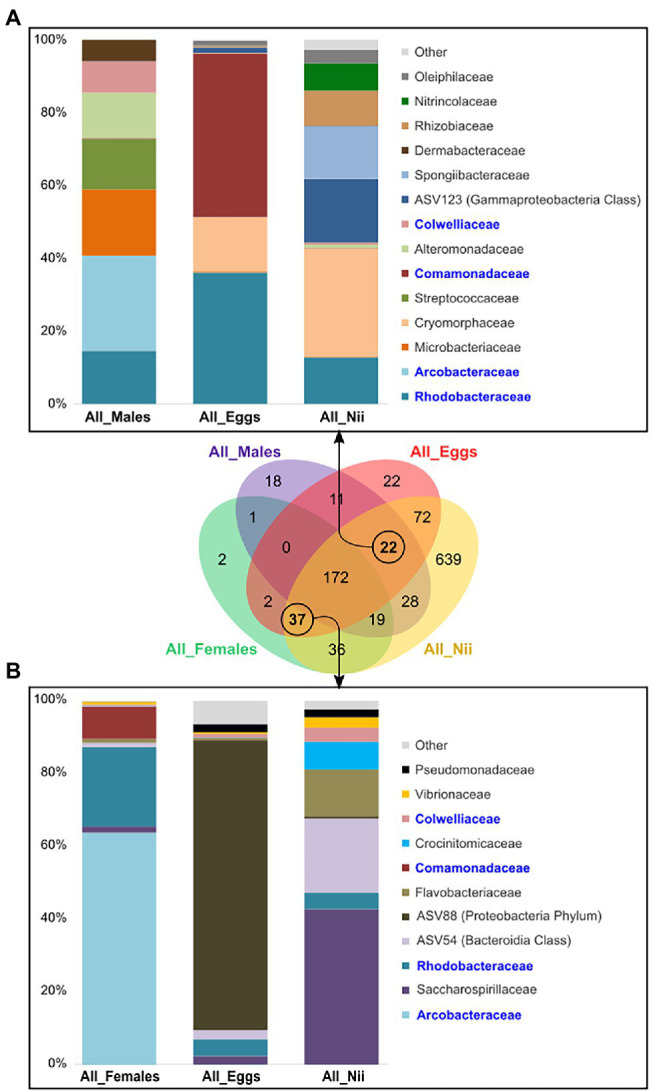
Shared bacterial lineages among the eggs, the nauplii, and the breeders. Top bacterial families with a total relative abundance higher than 1% shared among **(A)** the male, the egg and the nauplius (nii) samples (22 ASVs) and **(B)** the female, the egg and the nauplius (nii) samples (37 ASVs). The bacterial families in blue are the bacterial families shared between the two histograms which were constructed by averaging the triplicates for each sample type.

The eggs and the nauplii also shared specific ASVs with the female and the male samples taken separately. Indeed, the male, the egg, and the nauplius samples shared 22 ASVs, whereas 37 ASVs were uniquely found in the female reproductive organs, the eggs, and the nauplii. When considering the ASVs common to the males and their offspring, 14 dominant families were highlighted (Figure 5A). A total of 11 dominant families were observed when analyzing the ASVs shared by the females, the eggs, and the nauplii (Figure 5B). Even though different ASVs were involved in each group, four common families were found in the two constructed histograms: *Arcobacteraceae*, *Rhodobacteraceae*, *Comamonadaceae*, and *Colwelliaceae*. In both cases, the abundance profiles of the breeders, the eggs and the nauplii were very different and sometimes even opposite. Indeed, the breeders showed higher abundances of *Arcobacteraceae* than their offspring. On the contrary, the eggs and the nauplii respectively displayed larger proportions of *Comamonadaceae* and *Spongiibacteraceae* compared to the male samples. Furthermore, *Cryomorphaceae* were less abundant in the male reproductive organs than in the egg and the larval stages. Finally, *Saccharospirillaceae* were less highlighted in the ovarian tissues than in the nauplii.

### Shared Bacterial Lineages Among the Eggs and the Nauplii

**Figure 6:**
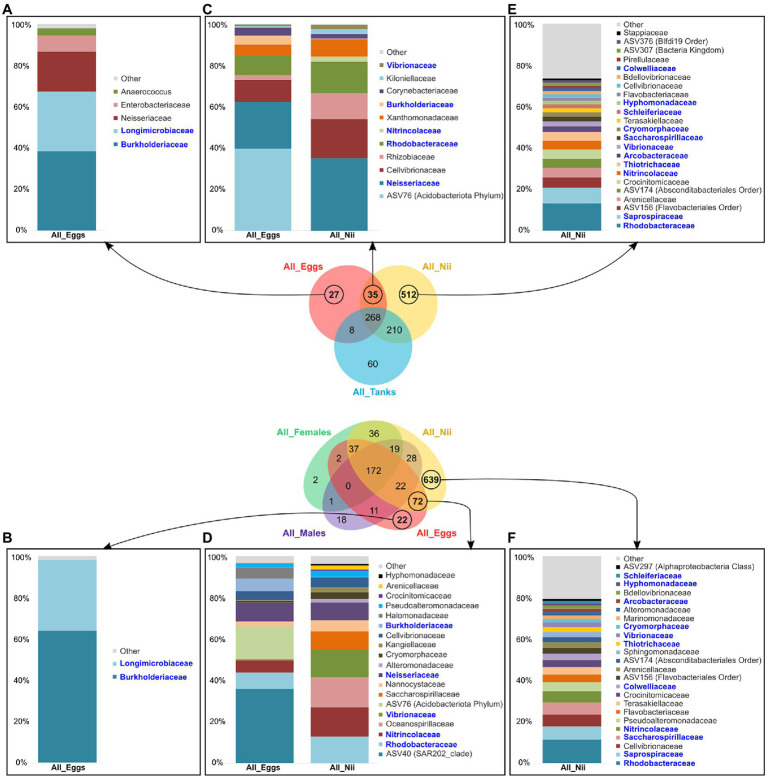
Specific and shared bacterial lineages among the eggs and the nauplii. Top bacterial families with a total relative abundance higher than 1% **(A)** specifically found in the eggs in Venn1 (27 ASVs) and **(B)** in Venn2 (22 ASVs), **(C)** shared between the egg and the nauplius (nii) samples in Venn1 (35 ASVs) and **(D)** in Venn 2 (72 ASVs), **(E)** specifically associated with the nauplii (nii) in Venn1 (512 ASVs), and **(F)** in Venn2 (639 ASVs). The bacterial families in blue are the bacterial families shared between histograms which were constructed by averaging the triplicates for each sample type.

The egg and the nauplius samples showed specific and shared ASVs in both global Venn diagrams. Indeed, the egg samples displayed a total of 27 specific ASVs in Figure 6A, whereas 22 specific ASVs were highlighted in Figure 6B. Analyzing the dominant bacterial families from these ASVs, *Longimicrobiaceae* and *Burkholderiaceae* appeared to be highly abundant in both groups. The eggs also shared ASVs with the nauplii in the two Venn diagrams. In the first Venn diagram, 35 ASVs were common to the both sample types (Figure 6C), while 72 ASVs were highlighted in the second Venn diagram (Figure 6D). The dominant bacterial families highlighted from these ASV groups showed five consistent families: *Rhodobacteraceae*, *Nitrincolaceae*, *Vibrionaceae*, *Neisseraceae*, and *Burkholderiaceae*. In both cases, abundance profiles differed between the two sample types as bacterial lineages abundant in the eggs decreased in the nauplii and vice versa. Finally, the nauplius samples showed a large amount of specific ASVs in both Venn diagrams (512 and 639 ASVs; Figures 6E,F). These groups of ASVs each showed various dominant bacterial families with homogeneous abundances. However, 11 families were common to both histograms: *Rhodobacteraceae*, *Saprospiraceae*, *Saccharospirillaceae*, *Nitrincolaceae*, *Colwelliaceae*, *Thiotrichaceae*, *Vibrionaceae*, *Cryomorphaceae*, *Arcobacteraceae*, *Hyphomonadaceae*, and *Schleiferiaceae*.

## Discussion

### Total Microbial Compositions Throughout all the Samples

The tank water samples were majorly composed of *Gammaproteobacteria* as well as *Alphaproteobacteria* and *Bacteroidia* (Figure 2B). These three major bacterial classes had been previously identified in water samples collected from the water reservoirs of the Saint Vincent experimental shrimp hatchery in October and November 2018 and February 2019. However, these reservoir samples also showed high abundances of *Acidimicrobiia*, *Cyanobacteriia*, and *Planctomycetes* (Giraud et al., 2021). Thus, differences in total microbial compositions were observed between the water in the tanks and the water used to fill the tanks. As samples were collected at different periods and seasons, these differences could be explained by both seasonal and annual variations impacting the bacterial composition of the natural seawater in the Saint Vincent Bay. Such variations have been shown in the coastal seawaters surrounding the subtropical Xiamen Island in China, where *Proteobacteria*, *Bacteroidetes*, *Cyanobacteria*, and *Firmicutes* dominated all samples even though more than 400 bacterial groups were identified as unique to at least one season (Wang et al., 2019). Similar conclusions were drawn from a 5-year study focusing on the bacterial genera associated with seawater from the Ofunato Bay in Japan (Kobiyama et al., 2021). Beyond seasonal variations, another hypothesis can be made in order to explain the differences observed in total microbial compositions between the tanks and the reservoirs. Indeed, unlike our previous study, water from the hatchery tanks was sampled after EDTA addition. A recent research has shown that microbial communities can be influenced by different EDTA concentrations in different soils (Wei et al., 2020) making it possible for EDTA to also influence microorganisms associated with seawater. In any case, tank water samples were dominated by *Gammaproteobacteria*, *Alphaproteobacteria*, and *Bacteroidia* which are common and important bacterial classes in various seawaters ranging from pelagic to benthic environments (Giovannoni and Stingl, 2005; Zinger et al., 2011). These bacterial classes were also very abundant in the nauplius samples which showed similar total microbial compositions than the water samples (Figure 2B). This was expected as these microbial communities have been shown in larvae and adults of different shrimp species under farmed conditions (Rungrassamee et al., 2013; Zheng et al., 2017). Furthermore, the rearing environment is known to be a key factor in the structure of the microbiota associated with fish larvae (Giatsis et al., 2015; Vadstein et al., 2018). Contrary to what we had shown previously, in this study, the nauplius samples grouped with the tank water samples and not with the eggs (Giraud et al., 2021). Indeed, when considering the microbial communities associated with the reproductive organs of the breeders, egg samples clustered with the male and the female samples (Figure 2A). In these samples, except for the Egg_3 and Female_2 samples which were clustered in a separate subgroup (Figure 2A), inter-individual variability was greater as abundances of *Clostridia*, *Bacilli*, and *Actinobacteria* varied (Figure 2B). High variability among the microbiotas of reproductive organs have already been shown in insects. Indeed, a study focusing on the microbiotas associated to different organs of nine mosquito species has shown that even though organs share core microbiotas, a tissue-specific tropism exists (Mancini et al., 2018). These results showed that the nauplii and the water from the hatchery tanks had similar microbial compositions, while the eggs were closer to the breeders. This suggested that the microbiota of the eggs could be under the influence of vertically transmitted microorganisms from the breeders and that after hatching, the microbiota of the nauplii could then be shaped by the rearing environment. In order to confirm our hypotheses, Venn diagrams were constructed to investigate the specific and shared bacterial communities among all the considered sample types (Supplementary Figure S1; Figure 3).

### Influence of the Rearing Environment on the Microbiota Associated With the Animals

Analyzing the constructed Venn diagrams, we were able to identify a core microbiota shared by the tank waters, the eggs and the nauplii and composed of 268 ASVs from which 11 dominant bacterial families were highlighted (Figure 4A). Out of these 11 families, 10 were also found in the core microbiota of the males, the females, the eggs, and the nauplii (Figure 4B): *Colwelliaceae*, *Alteromonadaceae*, *Vibrionaceae*, *Rhodobacteraceae*, *Saccharospirillaceae*, *Pseudoalteromonadaceae*, *Burkholderiaceae*, *Oceanosprilliaceae*, *Flavobacteriaceae*, and *Corynebacteriaceae*. *Colwelliaceae*, *Alteromonadaceae*, and *Pseudoalteromonadaceae* are all affiliated to the *Alteromonadales* order and the *Gammaproteobacteria* class. Members of these bacterial families are found in various environments ranging from soil and sediments to coastal and open seawaters. They can also be associated with organisms such as algae, fish, and marine invertebrates. This ability to colonize very diverse habitats can be explained by the production of various primary and secondary metabolites (hydrolytic enzymes, cyclic peptides, proteins, pigments, exopolymers, alkaloids…) which enable these microorganisms to decompose different organic compounds like hydrocarbons, lipids, proteins, and polysaccharides (Ivanova and Mikhailov, 2001; Bowman, 2014; Ivanova et al., 2014). *Vibrionaceae*, *Burkholderiaceae*, *Flavobacteriaceae*, *Corynebacteriaceae*, and *Rhodobacteraceae* are also known for their metabolic versatility and ecological diversity as they can be found in fresh and seawaters, soil, mud, plants, animals, and even humans (Riegel, 2006; Bernardet, 2015; Farmer and Janda, 2015; Garrity et al., 2015a,c). *Saccharospirillaceae* and *Oceanospirillaceae* are ubiquitous as well but are restricted to aquatic environments (Garrity et al., 2015b; Kevbrin et al., 2020). In 2011, research on the intestinal microbiota of the Chinese shrimp *Fenneropeaneus chinensis* highlighted four out of eight bacterial genera (*Vibrio*, *Loktanella*, *Roseobacter*, and *Flavobacterium*) affiliated to the *Vibrionaceae*, *Rhodobacteraceae*, and *Flavobacteriaceae* families (Liu et al., 2011). The three same bacterial families were also highlighted in a study focusing on the intestinal microbial communities of post-larvae and juveniles of the Pacific white shrimp *Litopenaeus vannamei*. The authors even suggested that *Flavobacteriaceae* and *Rhodobacteraceae* formed the core microbiota of all the samples they considered (Huang et al., 2016). More recently, in 2020, studies focusing on the microbiota associated with the larval stages of *L. vannamei* highlighted *Vibrionaceae*, *Pseudoalteromonadaceae*, *Oceanospirillaceae*, *Flavobacteriaceae*, *Colwelliaceae*, *Rhodobacteraceae*, and *Alteromonadaceae* in their samples (Wang et al., 2020a,b). The same bacterial families were highlighted in the eggs and the nauplii of *L. stylirotris* (Giraud et al., 2021). Thus, *Vibrionaceae*, *Rhodobacteraceae*, *Flavobacteriaceae*, *Pseudoalteromonadaceae*, *Oceanospirillaceae*, *Colwelliaceae*, and *Alteromonadaceae* were found in larvae and adults of different shrimp species. As these families were also associated with the tank water samples and as water was collected before transfer of the females into the hatchery tanks, we can hypothesize that some or all bacterial communities may be acquired from the rearing environment by the animals at larval or adult stages. As gut opening occurs after the nauplius stage in penaeid shrimps, water may modulate the epibiota of the larvae, while breeders could acquire microorganisms from the water and transmit them on to their offspring *via* a potential vertical transmission shaping the endobiota. In order to investigate this possible vertical transmission, we analyzed the ASVs shared among the larvae and the breeders taken separately.

### Arguments in Favor of a Potential Vertical Transmission

Even though the males were never in direct contact with their offspring, they shared 22 ASVs and 14 bacterial families (Figure 5A). The females, the eggs and the nauplii had 37 ASVs and 11 bacterial families in common (Figure 5B). Among these two sets of core bacterial families, *Arcobacteraceae*, *Rhodobacteraceae*, *Comamonadaceae*, and *Colwelliaceae* were found. *Arcobacteraceae* are members of the *Campilobacteria* class and have been found in the intestinal microbiota associated with juveniles of *L. vannamei* (Zeng et al., 2017). This bacterial clade has also been identified in the eggs of the vent shrimp *Rimicaris exoculata* (Guri et al., 2012; Methou et al., 2019). Vertical transmission of *Arcobacteraceae* has been proved in poultry and in pigs under farmed conditions (Collado and Figueras, 2011). *Comamonadaceae* belong to the *Burkholderiales* order and are thus metabolically diverse explaining why they are found in various environments such as soil, mud, and water in natural and anthropic environments (Willems and Gillis, 2015). Members of this bacterial family have been found in the discus fish *Symphysodon aequifasciata* in adults and their fry. The authors suggested that these microorganisms were vertically transferred from the parents to their offspring (Sylvain and Derome, 2017). *Comamonadaceae* have also been identified in the gut microbiota of *L. vannamei* at post-larval stage (Huang et al., 2016; Li et al., 2018). In order to explain the presence of these bacterial communities in the intestines of the post-larvae, Huang et al. (2016) hypothesized that these microorganisms were acquired from the *Artemia* larvae used for feeding. In our study, focus was made on the eggs and the first larval stages; thus, feeding could not explain the presence of *Comamonadaceae* in the eggs and the nauplii. However, they were found in the reproductive organs of the breeders. In the other mentioned studies, all bacterial strains were found in the intestinal microbiota as well. Relationships between the microbial communities of the gut and the reproductive organs are not well-documented but studies have established a link between the gut microbiota and the reproductive endocrine system in women. Indeed, disruption in the bacterial communities associated with intestines in women has been proved to cause reproductive diseases and syndromes. Overall, the gut microbiota is known to have various important effects on the intestines and to influence more distinct organs and molecular pathways in the human body (Qi et al., 2019, 2021). Taken together, these results suggest a potential vertical transmission of bacteria affiliated to the *Arcobacteraceae*, *Rhodobacteraceae*, *Comamonadaceae*, and *Colwelliaceae* families from the breeders to their offspring. However, further studies will be necessary in order to prove this possible vertical transmission. For example, in salmons, vertical transmission of the pathogenic bacteria *Piscirickettsia salmonis* was proved using indirect immunofluorescence technique and scanning electron microscopy (Larenas et al., 2003). However, this was possible for only one bacterial strain while we have highlighted several possible microbial families. Applying such technique in shrimps would require more investigations and important technical developments. Indeed, validating vertical transmission involves understanding the underlying cellular mechanisms. The development of specific probes could be worth considering for qualitative and quantitative tracking of specific bacterial strains (Lee et al., 2009). However, it is important to note that targeting certain microorganisms of interest is necessary.

### Undetermined Factors May Shape the Bacterial Communities Associated With the Eggs and the Nauplii

Our first results suggested that the environment and the breeders could shape the microbiota associated with the eggs and the early larval stages of *L. stylirostris*. However, as the eggs and the nauplii showed specific ASVs that were not highlighted in the tanks nor in the male and female reproductive organs, it seemed possible that other factors were involved. In both constructed Venn diagrams, ASVs which were only found in the egg samples were highlighted (Figures 6A,B). Only two dominant active bacterial families were highlighted from these ASVs: *Longimicrobiaceae* and *Burkholderiaceae*. Surprisingly, *Longimicrobiaceae* members (*Gemmatimonadota* phylum) are very common in soil and represent up to 2% of the soil microbial community (Bertrand et al., 2018). To our knowledge, these microorganisms have never been identified in marine seawaters or aquatic organisms. However, they have been found in the sediments of urbanized rivers (Ge et al., 2021). Adult shrimps are cultured in earthen ponds and their intestinal microbiota can share up to 80% ASVs with the sediments in their rearing environment (Hou et al., 2018). The potential influence of the gut microbiota has previously been discussed. However, *Longimicrobiaceae* were not highlighted in the reproductive organs of the breeders. As we studied active microbial communities, it is possible that this bacterial family was present and inactive in the breeders and became active when transmitted on to the eggs. Further investigations will be necessary to understand the role of *Longimicrobiaceae* in early larval stages of *L. stylirostris*. On the other hand, the presence of *Burkholderiaceae* was not surprising as they have been found in the intestines of post-larvae and juveniles of *L. vannamei* (Huang et al., 2016). As previously suggested for *Commamonadaceae*, *Burkholderiaceae* may be acquired at very early larval stages and may persist throughout the whole lifecycle of the shrimp. The egg samples also shared specific bacterial lineages with the nauplii in the Venn diagrams in Figures 6C,D. A total of five bacterial families were consistently found: *Rhodobacteraceae*, *Nitrincolaceae*, *Vibrionaceae*, *Neisseriaceae*, and *Burkholderiaceae*. *Rhodobacteraceae*, *Vibrionaceae*, and *Burkholderiaceae* were identified in all the samples but from different ASVs. Members of these families are able to produce extracellular proteins enabling them to colonize various habitats and to adapt, explaining why they were found in every compartment of our experiment. *Nitrincolaceae* has been identified in the gut microbiota of reared and wild salmons (Lavoie et al., 2021) as well as in the rearing water of sablefish larvae (Lee et al., 2021) and in *L. stylirostris* larvae (Giraud et al., 2021). The ability of the *Nitrincolaceae* members to colonize the intestines of marine species as well as their rearing environments may support our idea that some bacterial lineages could also be acquired from the environment. As previously suggested before for the breeders, environmental communities may activate when in contact with the larvae. Finally, nauplii displayed many specific ASVs that were not identified in the other sample types (Figures 6E,F). Considering the major active bacterial families identified from these ASVs, no family had a clear higher relative abundance. This was also illustrated with the analysis of the diversity index (Supplementary Figure S2). Indeed, the nauplius samples showed a significantly higher Chao1 index reflecting a higher richness of their microbial communities. However, no differences were observed for the Simpson index reflecting no change in evenness (Kim et al., 2017). This has already been reported in *L. vannamei* as the nauplii presented higher richness index than the later larval stages (zoea and mysis; Wang et al., 2020a). The increase of microbial richness associated with the nauplii was explained by yolk nutrient release and surface expansion at this stage. The authors suggested that as larvae grew and as their intestinal tract formed, food and environment tended to structure the gut microbiota leading to less richness. The authors also suggested that the microbial communities associated with the nauplii originated from the eggs. However, our results suggest otherwise. As previously mentioned, these communities may have been acquired from the water and/or the breeders and activated as the environmental conditions were more favorable. Other factors could also influence the structure of the microbial communities associated with the larvae such as competition between microorganisms, genetic background, larval ontogenesis, and host associated selection pressure (Vadstein et al., 2018; Sun and Xu, 2021). This is supported by the abundance difference of the microbial communities that were shared by the samples. Indeed, even when samples shared bacterial families, their relative abundances varied greatly supporting our theory that microbial communities may find more or less favorable conditions in each compartment. This has been previously shown during larval stages of *L. vannamei* where bacterial family abundances varied from one stage to another and where several stage-specific groups were highlighted (Zheng et al., 2017; Wang et al., 2020b). These results suggest that even though environment and breeders could influence the microbiota of the early larval stages, some unconsidered factors could also structure the microbial communities associated with the eggs and the nauplii.

## Conclusion

**Figure 7:**
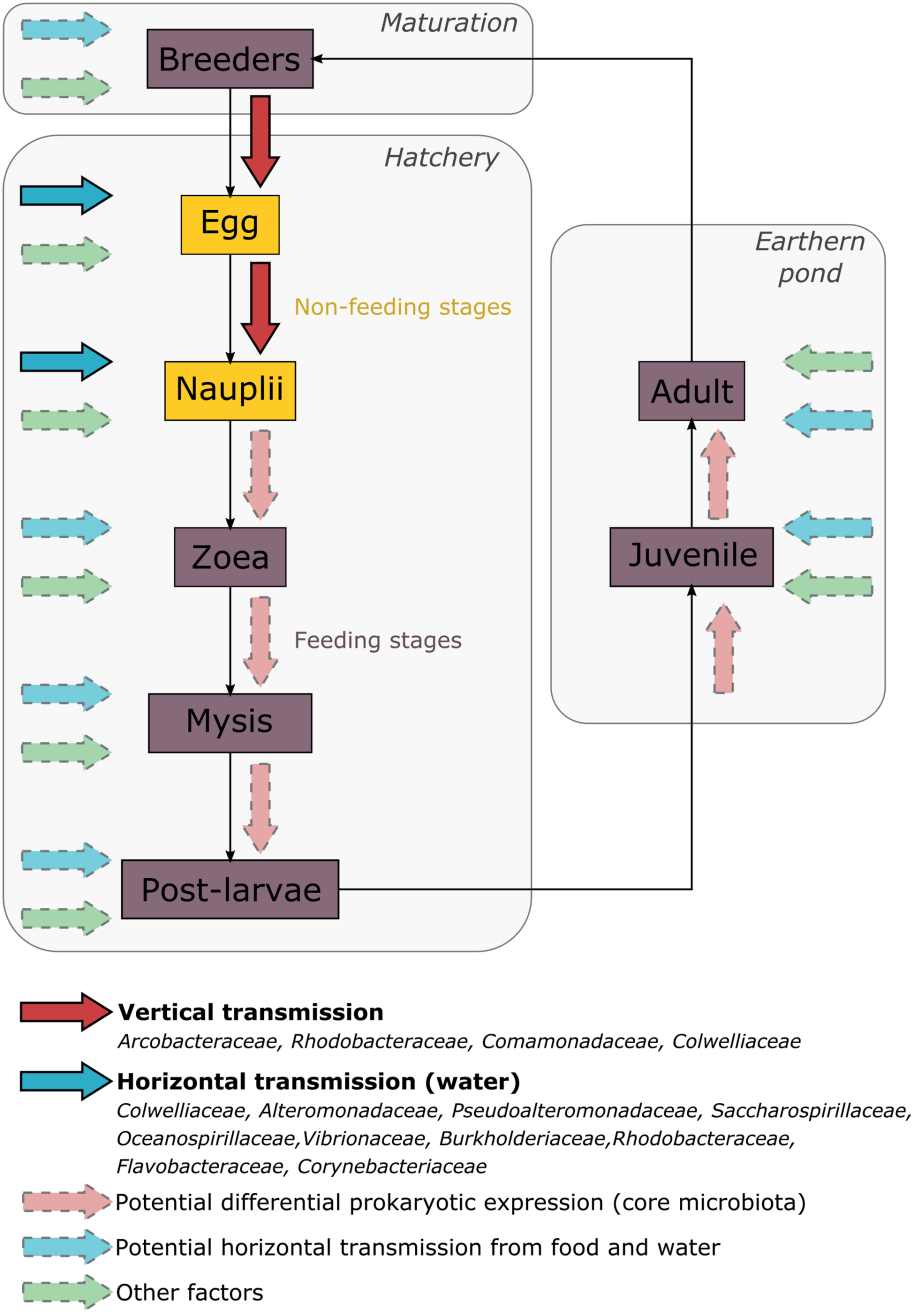
Schematic representation of the factors influencing the microbiota of the Pacific blue shrimp at all developmental stages. The egg, nauplii, zoea, and mysis stages belong to the larval stages.

In conclusion, we highlighted an active core microbiota associated with all the considered samples. Indeed, the water filling the hatchery tanks, the eggs and the nauplii as well as the female and the male reproductive organs shared 10 bacterial families: *Colwelliaceae*, *Alteromonadaceae*, *Pseudoalteromonadaceae*, *Saccharospirillaceae*, *Oceanospirillaceae*, *Vibrionaceae*, *Burkholderiaceae*, *Rhodobacteraceae*, *Flavobacteraceae*, and *Corynebacteriaceae*. Several bacterial families were also shared only by the breeders and their offspring: *Arcobacteraceae*, *Rhodobacteraceae*, *Comamonadaceae*, and *Colwelliaceae*. Finally, the egg and the nauplius samples showed unique bacterial families that were not identified in the other sample types. Taken together, our results suggest that the early larval stages of *L. stylirostris* are shaped by their environment and by their breeders. Some bacterial lineages may be horizontally acquired from the rearing water through adsorption and adhesion process at the surface of the larvae (Hansen and Olafsen, 1999). As some microorganisms can enter the larvae before mouth opening, these early acquired communities could shape the epibiota as well as the endobiota of the eggs and the nauplii. However, we also showed that some bacterial strains were shared with the breeders suggesting a potential vertical transmission affecting the structure of the larval microbiota. Furthermore, we highlighted bacterial families that were uniquely associated with the eggs and the nauplii suggesting that some unconsidered factors were involved (genetic background, host selection pressure, and so on). Thus, microbial communities associated with the eggs and the nauplii result in dynamic interactions that may persist through the whole larval cycle and even through the whole life cycle of the shrimp. Further investigations will be necessary in order to understand the evolution of the active microbial communities associated with later life stages as our results suggest that some bacterial lineages in shrimp larvae cultured in hatcheries may be determined way in advance by the breeders and their rearing environment. In addition, during further larval development, other factors like food could come into play to shape the microbiota (Figure 7). In aquaculture, microbial management of the rearing water is an important concern (Rurangwa and Verdegem, 2015). However, our study suggests that the microbiota associated with the animals can also be modulated by other factors leading to rethink microbial management of the whole rearing process.

## Data Availability Statement

The datasets presented in this study can be found in online repositories. The names of the repository/repositories and accession number(s) can be found in the article/Supplementary Material.

## Author Contributions

CG, NC, and NW conceived and designed the experiment. CG, NC, J-SL, DP, and NW performed the experiment and acquired the data. CG and NC analyzed the data, prepared the figures, and drafted the manuscript. VB, DP, NS-F, and NW reviewed the draft. All authors contributed to the article and approved the submitted version.

## Funding

This work was supported by the RESSAC project (LEAD-NC, Ifremer New-Caledonia) within the framework agreement with the New Caledonian Provinces and Government and by the Pacific Doctoral School.

## Conflict of Interest

The authors declare that the research was conducted in the absence of any commercial or financial relationships that could be construed as a potential conflict of interest.

## Publisher’s Note

All claims expressed in this article are solely those of the authors and do not necessarily represent those of their affiliated organizations, or those of the publisher, the editors and the reviewers. Any product that may be evaluated in this article, or claim that may be made by its manufacturer, is not guaranteed or endorsed by the publisher.

## Footnotes

^1^ http://jvenn.toulouse.inra.fr/app/example.html

## Supplementary Material

Supplementary Figure S1 Venn diagrams of each sample type. Specific and shared ASVs among all **(A)** the tank water samples, **(B)** the female reproductive organ samples, **(C)** the male reproductive organ samples, **(D)** the egg samples, and **(E)** the nauplius (nii) samples. For all the Venn diagrams, each ellipse color corresponds to a two-parent family. All samples from the first family are represented in blue. All samples from the second family are represented in yellow. All samples from the third family are represented in red. In both Venn diagrams, numbers noted in the overlapping areas correspond to the number of shared ASVs among samples while numbers noted outside of the overlapping areas correspond to the numbers of specific ASVs associated with each sample.

Supplementary Figure S2 Alpha diversity index of all samples. **(A)** Chao1 and **(B)** Simpson index for all the tank waters, the egg, the nauplius (nii), the female and the male samples. Significant differences (value of *p* < 0.05) highlighted by a Kruskal-Wallis and Dunn test are represented by *.
