# Supplementary figures and images for: The Active Microbiota of the Eggs and the Nauplii of the Pacific Blue Shrimp Litopenaeus stylirostris Partially Shaped by a Potential Vertical Transmission
Source: Front Microbiol. 2022 May 12;13:886752. doi: 10.3389/fmicb.2022.886752 (PMC9133551; doi:10.3389/fmicb.2022.886752)

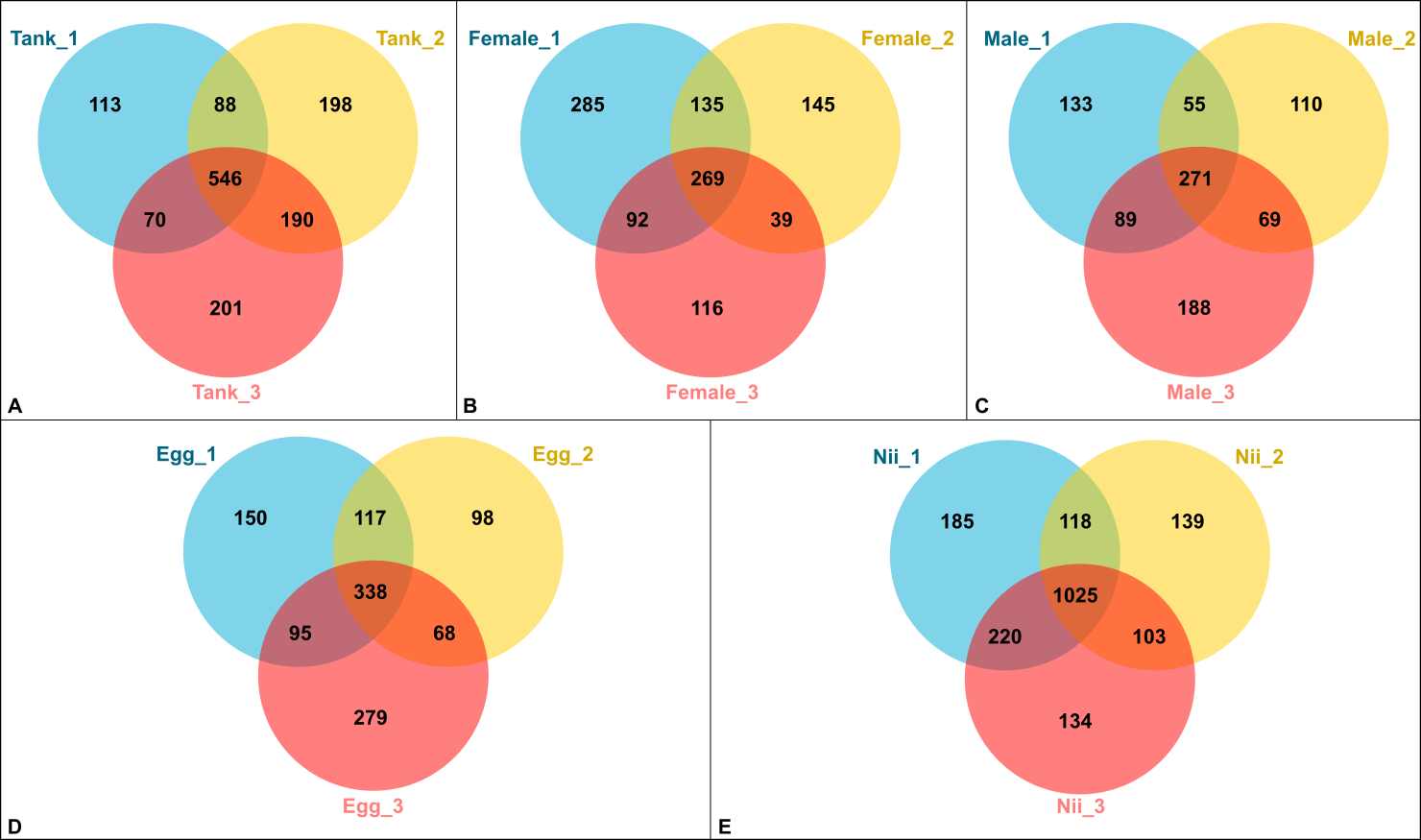

Supplement: Supplementary Figure S1 — Venn diagrams of each sample type. Specific and shared ASVs among all (A) the tank water samples, (B) the female reproductive organ samples, (C) the male reproductive organ samples, (D) the egg samples, and (E) the nauplius (nii) samples. For all the Venn diagrams, each ellipse color corresponds to a two-parent family. All samples from the first family are represented in blue. All samples from the second family are represented in yellow. All samples from the third family are represented in red. In both Venn diagrams, numbers noted in the overlapping areas correspond to the number of shared ASVs among samples while numbers noted outside of the overlapping areas correspond to the numbers of specific ASVs associated with each sample. [file Image_1.TIF]

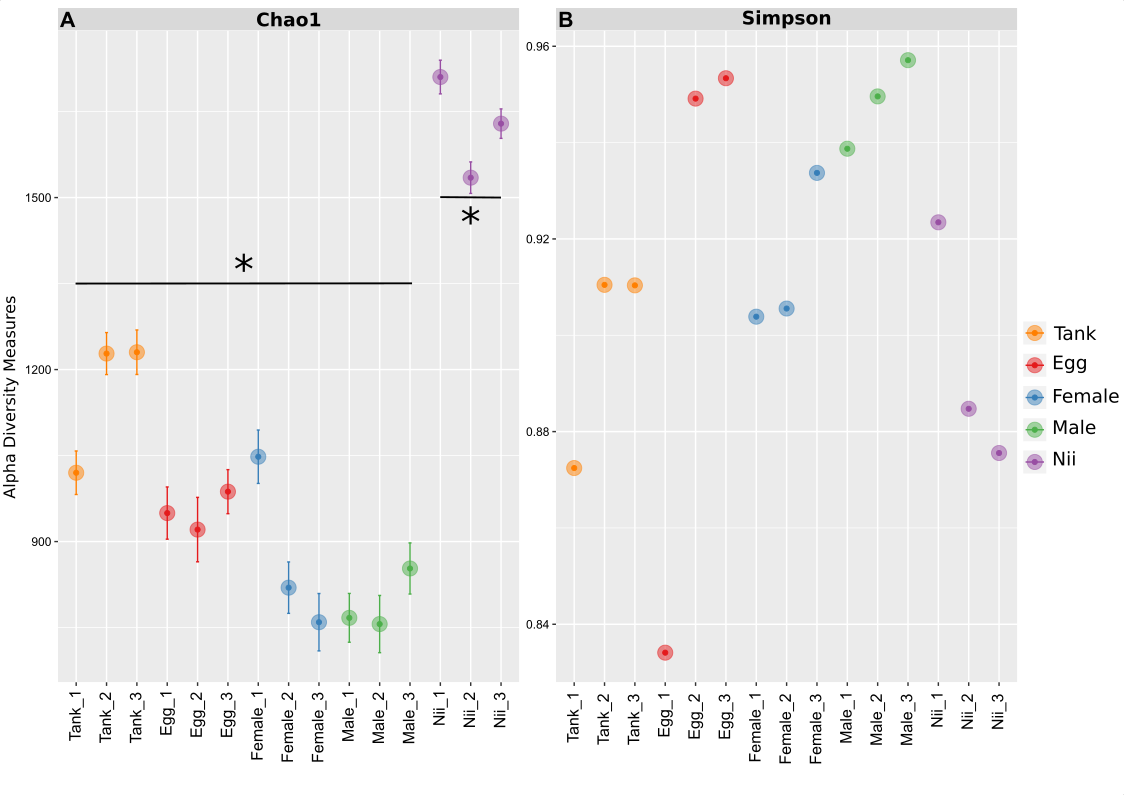

Supplement: Supplementary Figure S2 — Alpha diversity index of all samples. (A) Chao1 and (B) Simpson index for all the tank waters, the egg, the nauplius (nii), the female and the male samples. Significant differences (value of p < 0.05) highlighted by a Kruskal-Wallis and Dunn test are represented by *. [file Image_2.TIF]
